# Surgical Outcomes and Prognostic Factors of T4 Gastric Cancer Patients without Distant Metastasis

**DOI:** 10.1371/journal.pone.0107061

**Published:** 2014-09-11

**Authors:** Ming-zhe Li, Liang Deng, Jing-jing Wang, Long-bin Xiao, Wen-hui Wu, Shi-bin Yang, Wen-feng Li

**Affiliations:** 1 Department of Gastrointestinal and Pancreatic Surgery, The First Affiliated Hospital, Sun Yat-sen University, Guangzhou, China; 2 Department of General Surgery I, the Eastern Hospital of the First Affiliated Hospital, Sun Yat-sen University, Guangzhou, China; 3 Department of laboratory, Hexian Memory Hospital of Panyu District, Guangzhou, China; H. Lee Moffitt Cancer Center & Research Institute, United States of America

## Abstract

**Objective:**

To evaluate surgical outcomes and prognostic factors for T4 gastric cancer treated with curative resection.

**Methods:**

Between January 1994 and December 2008, 94 patients diagnosed with histological T4 gastric carcinoma and treated with curative resection were recruited. Patient characteristics, surgical complications, survival, and prognostic factors were analyzed.

**Results:**

Postoperative morbidity and mortality were 18.1% and 2.1%, respectively. Multivariate analysis indicated lymph node metastasis (hazard ratio, 2.496; 95% confidence interval, 1.218–5.115; p = 0.012) was independent prognostic factor.

**Conclusions:**

For patients with T4 gastric cancer, lymph node metastasis was associated with poorer survival. Neoadjuvant chemotherapy or aggressive adjuvant chemotherapy after radical resection was strongly recommended for these patients.

## Introduction

Although radical resection had been proved to be the most important indicator of long-term survival for patients with gastric cancer, curative resection for locally advanced gastric cancer, defined as T4 in which the tumor perforates serosa (T4a) or invades adjacent structures (T4b), was associated with increased postoperative morbidity and mortality. [1] With improved surgical technique and early detection of gastric cancer, the prognosis of gastric cancer patients has been gradually improved. However, the prognosis of patients with T4 gastric carcinoma remained poor. So it is essential to clarify the incidence of postoperative morbidity and mortality in T4 gastric cancer patients who undergo curative operations, and to determine the prognostic factors in such populations. In the present study, we retrospectively evaluated surgical outcomes and prognostic factors for T4 gastric cancer treated with curative resection.

## Patients and Methods

This study was approved by the ethic committee of the First Affiliated Hospital of Sun Yat-sen University. Patient information was anonymized and de-identified prior to analysis. Between January 1994 and December 2008, a total of 1249 patients with gastric cancer underwent gastrectomy with curative-intent at Department of Gastrointestinal and Pancreatic Surgery, the First Affiliated Hospital, Sun Yat-sen University. Of these, 132 patients were diagnosed as histological T4 gastric carcinoma, including 94 patients (71.2%) treated with curative resection and 38 patients treated (28.8%) with non-curative resection (R1 or R2 resections). Standard D2 lymph node dissections or D2 plus para-aortic lymph node dissections were performed in these patients with curative intent. A distal subtotal gastrectomy (SG) or total gastrectomy (TG) was performed depending on the location of the primary tumor. The curative (R0) resection was defined as the complete removal of cancer tissue with no residual tumor macroscopically or microscopically and no evidence of distant metastasis. Patients with metastatic disease who had undergone palliative resection were excluded. A doctor specialized in chemotherapy in our institution determined which patients received adjuvant therapy and the treatment protocols. The patients in this series did not receive neoadjuvant treatment.

Postoperative mortality was defined as deaths within 30 days after the surgery. Surgical morbidity was defined as any complication that occurred in the 30-day postoperative period. The complications were classified according to the Clavien-Dindo classification. [2] The basic monitoring, oral antibiotics, bowel rest, or supportive care were required for Grade I complication. The intravenous medication (antibiotics), transfusions, chest tubes, prolonged tube feedings, or total parenteral nutrition were required for Grade II complication. The interventional radiology, reoperation, intensive care unit admission, intubation, or bronchoscopy were required for Grade III complication. Grade IV complication resulted in permanent disability (renal failure requiring dialysis) or organ resection. Grade V complication resulted in the patient's death.

Clinicopathological data were obtained from a prospectively constructed medical database. Survival data were obtained from outpatient clinical visits, letter interviews or telephone interviews. Survival duration was calculated from the time of surgery to death or the last follow-up date.

Statistical analysis was performed using The SPSS program version 13.0 for Windows (SPSS Inc, Chicago, IL, USA). All categorical data were presented as rate and continuous data were expressed as mean ±standard deviation (SD). Survival was calculated using the Kaplan-Meier method. The statistical significance was assessed by the log-rank test. Factors that were deemed of potential importance on the univariate analysis (p<0.05) were included in the multivariate analysis by Cox regression. Hazard ratios (HR) with 95% confidence intervals (CI) were obtained as a measurement of association. P value <0.05 was considered statistically significant.

## Results

### Clinicopathologic characteristics

Ninety four patients diagnosed as histological T4 gastric carcinoma and treated with curative resection were selected for this study. Clinicopathological features of the patients were summarized in **Table 1**. The patients were comprised of 67 men (71.3%) and 17 women (28.7%) aged 31 to 75 years(mean±SD, 58.6±13.3 years). The mean diameter of the tumors was 73 mm (SD, 41 mm). The most common site of the primary lesion was positioned in the proximal stomach (36.2%). Twenty-seven patients (28.7%) underwent distal subtotal gastrectomy and sixty-seven (71.3%) underwent total gastrectomy. The majority of the cancers (78.7%) were poorly differentiated. Most patients (85.1%) showed lymph node involvement.

**Table 1:** Clinicopathologic characteristics of 94 Patients.

Clinical variable	Mean or patients	SD or Percent
***Age(y)***	58.6	13.3
***Gender***		
Male	67	71.3%
Female	17	28.7%
***Tumor diameter (mm)***	73	41
***Tumor location***		
Proximal	34	36.2%
Middle	19	20.2%
Distal	29	30.8%
Whole	12	12.8%
***Operation type***		
Subtotal gastrectomy	27	28.7%
Total gastrectomy	67	71.3%
***Borrmann type***		
I and II	19	20.2%
III	55	58.5%
IV	20	21.3%
***Histologic type***		
Well differentiated	20	21.3%
Poor-undifferentiated	74	78.7%
***Lymph node metastasis***		
Absence	14	14.9%
Presence	80	85.1%

SD: standard deviation.

Thirty-nine patients were included in the stage T4a group and 55 cases in the stage T4b group, according to TNM classification. [3] On histological examination, it was found that T4b gastric carcinomas exhibited invasions to the pancreas in 25 patients, the transverse colon in 17 patients, the spleen in 9 patients, the liver in 5 patients, the diaphragm in 3 patients, and gallbladder in 2 patient. Six patients had two organ invaded. Fourty-one patients (43.6%) postoperatively received CapeOX chemotherapy (capecitabine 1000 mg/m^2^ twice daily on days 1–14 and oxaliplatin 130 mg/m^2^ on day 1), 34 patients (36.2%) received FOLFOX chemotherapy (oxaliplatin 85 mg/m^2^ on day 1, L-leucovorin 200 mg/m^2^ on day 1, 400 mg/m^2^ bolus fluorouracil, and 2400 mg/m^2^ infusional fluorouracil on day 1–2) and 19 patients (20.2%) received SOX chemotherapy (S-1 40 mg/m^2^ twice daily on days 1–14 and oxaliplatin 130 mg/m^2^ on day 1). The patients in this series did not receive neoadjuvant treatment.

### Postoperative complications

The median length of stay was 26 days (range, 18–71days). A total of 17 patients (18.1%) had postoperative complications. These complications were listed in **Table 2**. Wound infection was the most frequent complication, occurring in 5 patients (5.3%). When complications were classified according to Clavien-Dindo classification, grade I complications were seen in 5 (5.3%) patients, grade II complications were seen in 8 (8.5%), grade III complications were seen in 1 (1.1%), grade IV complications were seen in 1 (1.1%), and grade V complications (mortality) were seen in 2 (2.1%) patients.

**Table 2:** Postoperative complications.

	Patients(n)	Percent (%)
Complications	17	18.1
Abdominal abscess	1	1.1
Pancreatic fistula	3	3.2
Anastomosis leakage	2	2.1
Pulmonary complication	3	3.2
Wound infection	5	5.3
Intra-abdominal hemorrhage	2	2.1
Gastrointestinal hemorrhage	1	1.1
Clavien-Dindo classification		
grade I	5	5.3
grade II	8	8.5
grade III	1	1.1
grade IV	1	1.1
grade V (mortality)	2	2.1

### Survival analysis

**Figure 1:**
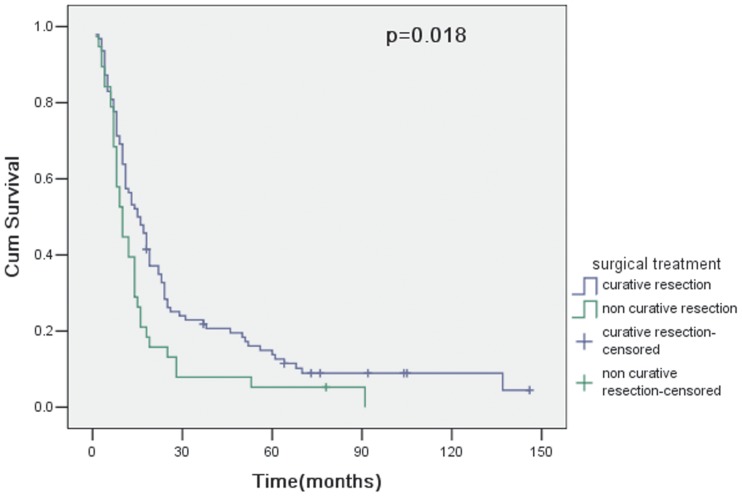
Cumulative survival rates in relation to surgical treatment, together with the p value from the log-rank test.

**Figure 2:**
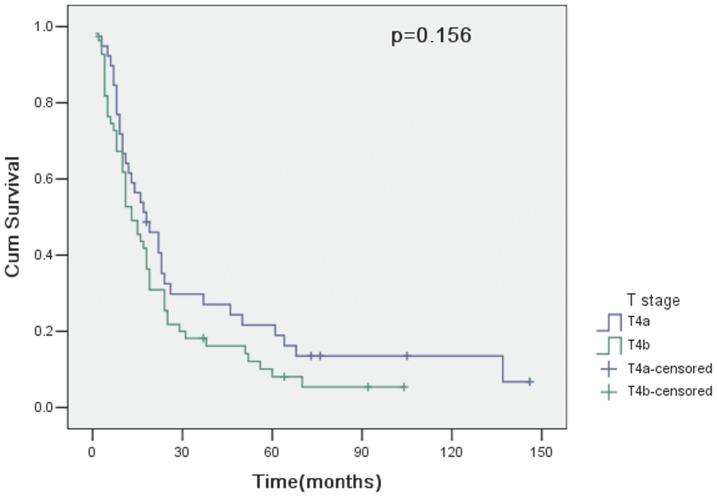
Cumulative survival rates in relation to T stage, together with the p value from the log-rank test.

**Figure 3:**
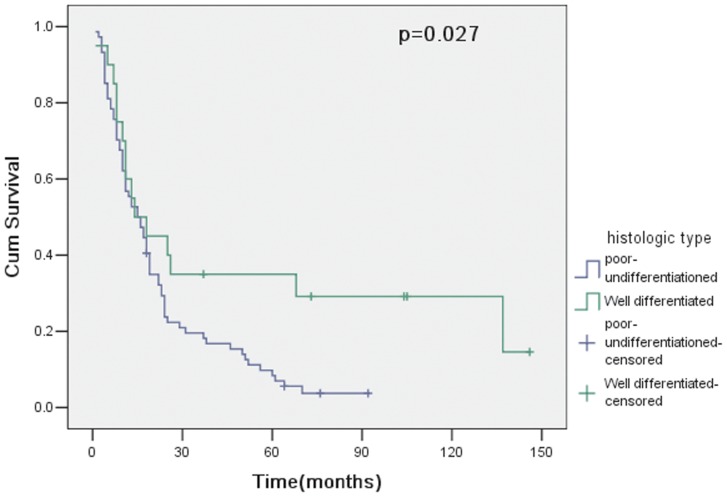
Cumulative survival rates in relation to histologic type, together with the p value from the log-rank test.

**Figure 4:**
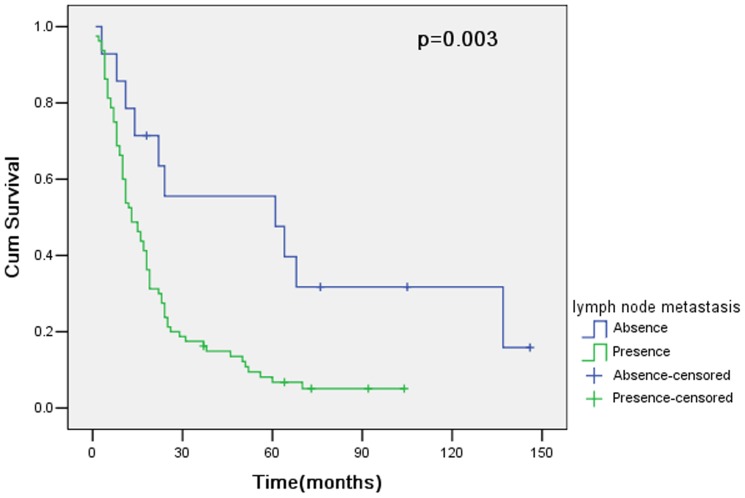
Cumulative survival rates in relation to lymph node metastasis, together with the p value from the log-rank test.

The overall survival of 94 patients with curative resection (R0) was 56.4% at 1 year, 22.9% at 3 years and 13.8% at 5 years. However, for 38 patients undergoing noncurative resection (R1 or R2), the overall survival was 39.5% at 1 year, 7.9% at 3 years and 5.3% at 5 years. Therefore, curative resection had a statistically significant influence on survival (p = 0.018; Fig. 1). In 94 patients with curative resection, survival curves for patients with pT4a and pT4b showed no significant difference between groups (p = 0.156; Fig. 2). The clinicopathologic variables evaluated in the univariate analysis were listed in **Table 3**. Histologic type (p = 0.027; **Fig. 3**) and lymph node metastasis (p = 0.003; **Fig. 4**) were associated with survival by univariate analysis. While only lymph node metastasis (hazard ratio, 2.496; 95% confidence interval, 1.218–5.115; p = 0.012) was identified as independent prognostic factors for long-term survival by multivariate analysis (**Table 4**).

**Table 3:** Univariate analysis of risk factors for patients with T4 gastric cancer.

Variables	3-yr survival rate (%)	5-yr survival rate (%)	Log-rank p
***Age(y)***			
<65	25.1	15.0	0.229
≥65	17.9	10.7	
***Gender***			
Male	20.2	15.5	0.533
Female	29.6	4.3	
***Tumor diameter (cm)***			
≤7	25.0	17.3	0.275
>7	20.3	8.9	
***Operation type***			
Subtotal gastrectomy	29.6	12.7	0.498
Total gastrectomy	20.1	13.9	
***Borrmann type***			
I and II	21.1	10.5	0.424
III	27.3	18.2	
IV	11.3	0	
***Histologic type*** *			
Well differentiated	35.0	35.0	0.027
Poor-undifferentiated	19.6	8.4	
***Lymph node metastasis*** *			
Absence	43.0	35.8	0.003
Presence	20.2	12.1	

*p value <0.05.

**Table 4:** Multivariable Cox regression analysis of risk factors for patients with T4 gastric cancer.

Clinical variable	Model coefficient	HR	95% CI for HR	p value
***Histologic type***				
Well differentiated		1.000		
Poor-undifferentiated	0.460	1.584	0.876–2.864	0.128
***Lymph node metastasis***				
Absence		1.000		
Presence	0.915	2.496	1.218–5.115	0.012

HR: hazard ratio.

## Discussion

Gastric cancer was the second leading cause of cancer death worldwide. Although surgical results for early stage gastric carcinoma were satisfactory, locally advanced gastric cancer still had a poor prognosis due to simultaneous distant metastasis such as peritoneal seeding or liver metastasis. A certain number of patients with T4 gastric carcinoma without distant metastasis could survive curative resection and progress satisfactorily without tumor recurrence. However, the morbidity and mortality increased significantly after curative resection. Reported morbidity and mortality rates ranged from 11.8% to 90.5% and from 0 to 15%, respectively. [4]–[7] In the present study, the surgical morbidity and mortality rates were 18.1% and 2.1%, respectively, which were comparable to previous reports. Different complication rates might be owing to different population and definition of complications. In this study, we graded the complications according to the Clavien-Dindo classification so that the results were more accurate. Most of these complications were grade I and II, which mainly required only short-term simple medical treatment. On the basis of our data, the increased postoperative complications were acceptable and most of them were not serious. Hence, aggressive surgical approach including multiorgan resection was still recommended for T4 gastric tumors.

The reported incidence of delayed gastric emptying (DGE) after gastrectomy ranged from 3.2 to 6.9%. [8]–[10] In the present study, postoperative DGE was not found among the 94 patients treated with curative resection. This might be caused by many reasons. Firstly, the majority of the patients (67 patients) underwent total gastrectomy, and only a few patients (27 patients) underwent subtotal gastrectomy. Secondly, the definition of DGE was various in the literature. Bar-Natan et al defined DGE as the inability to eat a regular diet after 10 postoperative days. [8] Kim KH defined DGE by patients' symptoms of gastric fullness, nausea, vomiting, and simple abdomen X-ray. [10] In our department, DGE was defined as the inability to eat a fluid diet after 7 postoperative days, with the symptoms of gastric fullness, nausea, vomiting. This definition was relatively strict and therefore some patients with mild DGE might be missed. Thirdly, malnutrition was considered to be associated with the development of postoperative DGE. In our department, nutrition support treatment was applied for every patient with malnutrition, in order to make their adequate nutritional status before surgery.

The median length of stay was 26 days in our series. It was longer than other international series reported. This might be related to the Chinese medical system and health care delivery models. In china, community medical was underdeveloped. Before operation, patients needed to stay in hospital many days for treatment of basic diseases, like hypertension, diabetes mellitus, malnutrition, etc. On the other hand, without family doctors to provide follow-up treatment for patients, the discharge standard in China was stricter than other places. Therefore, the length of stay prolonged.

In our series, the 1-, 3-, and 5-year overall survival was 56.4%, 22.9%, and 13.8% respectively. Overall survival was a complex issue that could be influenced by many factors. Kunisaki found curability, mall tumor diameter and numbers of lymph node metastases were prognostic factors and suggested that curative resection should be performed for T4 gastric cancer with relatively small tumors and few lymph node metastases. [11] Histologic type as an independent prognostic factor for long-term survival had not been reported. Theoretically, poor differentiated cancer cells had more aggressive biological behavior which led to poor prognosis. Univariate analysis in our study indicated that histologic type was an independent prognostic factor for T4 gastric cancer. However, this result was not confirmed in multivariate analysis. Insufficient sample size might be an important reason for this result. Age and tumor sizes were also reported as independent poor prognostic factors. [12]–[16] However, they were not confirmed in the present study. More high-quality studies were needed to clarify these prognostic factors.

Lymph node metastasis was also reported as an important indicator of prognosis for T4 gastric cancer. [17], [18] In the present study, lymph node metastasis was identified as an independent prognostic factor by univariate analysis and multivariate analysis. Although standard D2 lymph node dissection was performed in our series, potential lymph node metastases might not been removed. Dikken et al. demonstrated that postoperative chemotherapy could improve survival. [19] Therefore, for T4 gastric cancer with lymph node metastasis, aggressive chemotherapy was recommended after curative resection. If lymph node metastasis could be diagnosed by computed tomography or endoscopic ultrasonography before surgery, neoadjuvant chemotherapy was also a good choice. Theoretically, neoadjuvant chemotherapy might be superior to postoperative chemotherapy. Firstly, neoadjuvant chemotherapy potentially led to downstaging of the tumor and might therefore substantially facilitate its complete resection. Secondly, neoadjuvant chemotherapy could eliminate systemic micrometastases and decrease distant recurrence. Thirdly, neoadjuvant chemotherapy could be used to assess tumor chemosensitivity to cytotoxic medications. The results of MAGIC trial showed that perioperative chemotherapy conferred a considerable survival benefit, extending the 5-year survival rate from 23to 36%. [20] Lordick also stated that the neoadjuvant treatment could improved the rate of R0 resection and overall survival. [21] Recently, there was still increasing evidence that patients with T4 gastric cancer could benefit from neoadjuvant chemotherapy. Several multicenter phase II studies from the East Asia explored the efficacy and safety of neoadjuvant chemotherapy for clinically serosa-positive (T4a/b) gastric cancer. [22], [23] Yoshikawa demonstrated that neoadjuvant chemotherapy followed by D2 or more extended gastrectomy resulted in an R0 resection rate of 78%, with a pathological response in 39%. Postoperative morbidity and mortality rates were 10.2 and 0%, respectively. [22] Hirakawa M reported that neoadjuvant chemotherapy for locally advanced resectable gastric cancer resulted in a high R0 resection rate of 90.7%, with a pathological response of 65.9%. There were no treatment-related deaths and no major surgical complications. [23] Therefore, neoadjuvant therapy in T4 gastric cancer patients with lymph node metastasis was strongly recommended.

## Conclusions

For patients with T4 gastric cancer, lymph node metastasis was associated with poorer survival. Neoadjuvant chemotherapy or aggressive adjuvant chemotherapy after radical resection was strongly recommended for these patients.
